# A Performance Evaluation of Flexible Thermoelectric Modules for Application in a Cooling Garment

**DOI:** 10.3390/ma18030633

**Published:** 2025-01-30

**Authors:** Anna Dąbrowska, Monika Kobus, Łukasz Starzak, Bartosz Pękosławski, Paulina Łataś

**Affiliations:** 1 Department of Personal Protective Equipment, Central Institute for Labour Protection—National Research Institute, Wierzbowa 48, 90-133 Lodz, Poland; mokob@ciop.lodz.pl; 2 Department of Microelectronics and Computer Science, Lodz University of Technology, Wólczańska 221 Building B18, 93-005 Lodz, Poland; lukasz.starzak@p.lodz.pl (Ł.S.); bartosz.pekoslawski@p.lodz.pl (B.P.); 3 Institute of Materials Science and Engineering, Lodz University of Technology, Stefanowskiego 1/15, 90-537 Lodz, Poland

**Keywords:** cooling garment, thermoelectric cooling, Peltier effect, TEM

## Abstract

In recent years, significant progress in thermoelectric module (TEM) technology has been achieved in terms of both flexibility and efficiency. This has created great application potential for it, including in cooling garments. In this paper, the results from performance tests of six selected flexible TEMs are presented and discussed in terms of their applicability in a cooling garment. For this purpose, a special testing methodology was adopted that included the use of a skin model located in a microclimate chamber that allowed the analysis of the absorbed heat flow rate from the cold side of a TEM. In addition, electrical parameters were measured in order to calculate the coefficient of performance for each of the evaluated TEMs. Based on these measurements, the TEMs were compared in terms of the cold-side heat flow rate and the number of modules needed to achieve a given heat flow rate or total cooling surface area. The best results were achieved for the TEM with dimensions of 85 mm × 68 mm × 6 mm, for which a maximum heat flow rate of 1.39 W was achieved with an electrical supply power of 0.35 W. To achieve similar values with other evaluated TEMs, two to five modules would have to be applied.

## 1. Introduction

A need for novel, environmentally friendly thermal management and energy harvesting technologies has led to recent significant advancements in the field of thermoelectric modules (TEMs). Special attention has been paid to achieving the flexibility of TEMs without reducing their efficiency. According to Cao [1], a combination of several strategies has been adopted for this purpose, including various engineering, structuring and treatment methods [2,3,4], as well as material composition (organic or inorganic) [5]. Moreover, studies in the literature provide information on various forms of flexible TEMs. We can distinguish one-dimensional fiber-based flexible TEMs [6], two-dimensional film-based flexible TEMs [7] and three-dimensional bulk-based flexible TEMs [8], in which thermoelectric materials can be either rigid or flexible [9,10], but their substrates are always flexible [1,11]. The high flexibility achieved, combined with an increase in the efficiency of energy conversion from waste heat and the efficiency of heat transfer, has created great application potential for TEMs, including in cooling garments [1,12,13].

The interest in the application of TEMs in cooling garments has surely been driven by societal and industrial needs. According to a recent report published by the International Labour Organisation [14], at least 2.41 billion workers are exposed annually to excessive heat. Moreover, due to climate change, the thermal load at work is no longer related only to work in thermally constant environments (e.g., mining, glassworks, metallurgy industry), but also to outdoor work, especially in the summer season. Therefore, a need for new measures to overcome the issue of the negative influence of work in high air temperatures both indoor and outdoor has arisen. According to Amoadu et al. [15], there are several risk factors that may lead to heat-related illness such as gender, age, pre-shift dehydration, piece-rate payment, poor access to sanitation facilities, the use of inappropriate personal protective equipment, physically demanding work, a high workload, low job control and high temperatures. These factors should be taken into account especially in extreme environmental conditions, in which the risk of heat-related health consequences is high.

There are two kinds of cooling garments that are based on thermoelectric phenomena. In those that use direct thermoelectric cooling, excess heat from the body is absorbed through the conduction between the skin (which may be covered with an underwear layer) and the cold side of the TEM [16,17]. In the case of indirect cooling, TEMs are part of a water cooling system [18,19,20,21] or an air conditioning one [22]; thus, they cool a heat transfer medium.

An alternative to TEM-based personal cooling is the use of cooling liquids [23,24] or phase-change materials (PCMs) [25,26] alone. Cooling systems with TEMs (whether flexible or rigid) are in general much more lightweight (Table 1), which is especially important in wearable applications, and they can be easily integrated with garments, for instance, by manufacturing TEMs in a knitted form [27]. Moreover, TEM-based cooling systems can offer a similar or even higher reduction in skin temperature. As their electric power consumption is low (thanks to the high values of the coefficient of performance), the required operating time can be achieved using relatively compact and lightweight batteries [28,29]. Another advantage of many TEM-based systems is the lack of moving active components (such as fans or pumps) [16,17,27,28,29,30,31,32], which makes their operation noiseless and more reliable.

The application of thermoelectric modules for cooling purposes requires determining the operating parameters at which they achieve the greatest efficiency. The latter is influenced by factors such as, among others, the component materials, the temperature difference between the hot and cold side [33] and thus the method used to dissipate heat on the hot side of the module [34]. In order to evaluate the cooling performance of TEMs, several methods are currently used, none of them being standardized [13,35]. Some parameters are commonly used for characterizing TEMs, such as the figure of merit (ZT), the energy conversion coefficient η, the coefficient of performance (COP) or the maximum power density ω [1], although not all of them relate to cooling applications. In specific fields of the application of flexible TEMs, special testing and evaluation methodologies are adopted. In order to achieve a maximum cooling capacity, i.e., a maximum COP, the optimum supply current must be determined [36]. The significant impact of operating conditions on TEM operation and performance should also be taken into account. The aim of this work was to characterize flexible thermoelectric modules in terms of the highest performance obtained under specific operating conditions and to select the best-performing TEM for use in cooling garments.

## 2. Materials and Methods

### 2.1. Tested Object

**Figure 1:**
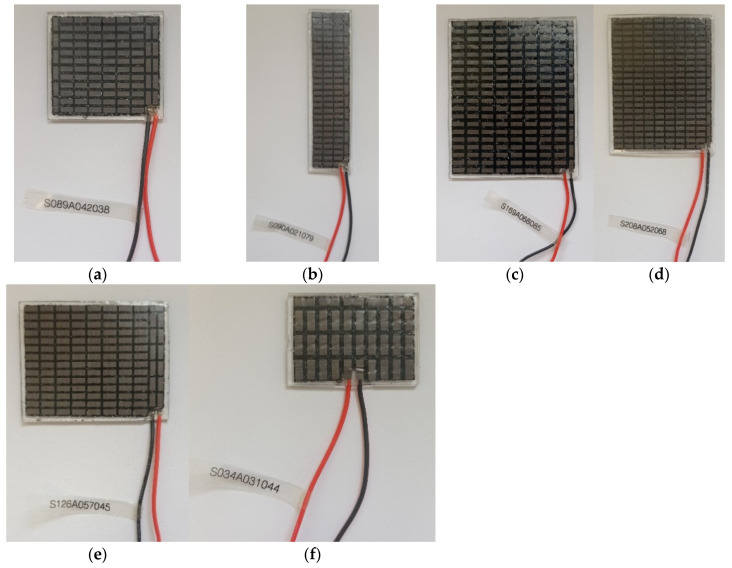
Photographs of thermoelectric modules selected for the tests: (**a**) S089A042038 (089), (**b**) S090A021079 (090), (**c**) S169A068085 (169), (**d**) S208A052068 (208), (**e**) S126A057045 (126) and (**f**) S034A031044 (034).

Six flexible thermoelectric modules offered by TEGway were selected for the laboratory tests, as presented in Figure 1. The basic specifications of each module are shown in Table 2, where

- S is the total surface area;
- I_rat_ is the maximum rated current;
- U_rat_ is the maximum rated voltage (occurring at I_max_);
- P_rat_ is the maximum rated electric power (supplied at I_max_), calculated as I_max_ × U_max_;
- Q_c(rat)_ is the maximum rated cold-side heat flow rate;
- q_c(rat)_ is the maximum rated cold-side heat flux, calculated as Q_c(rat)_/S.

The last two parameters are both related to the cooling performance of a TEM. However, it is the heat flux q_c_ that should be used to directly compare modules with different surface areas, as it is area-independent. An important parameter is also the COP. However, as argued in Section 4.2, its meaningful value cannot be determined from rated parameters. Finally, the surface area S will affect the number of necessary modules, an issue which will be discussed in Section 4.3.

The selected thermoelectric modules were tested in combination with a flame-retardant multi-shield fabric and a heat-dissipating module, the so-called heat sink. The multi-shield fabric had a thermal resistance of (0.016 ± 0.001) m^2^ × K/W and a water vapor resistance of (3.281 ± 0.171) m^2^ × Pa/W. Technical Absorbents SAF Fabric 2644 superabsorbent non-woven fabric was used for the heat sink. The non-woven fabric was trimmed on both sides with the multi-shield fabric. The sizes of the prepared heat sinks corresponded to those of the respective thermoelectric modules.

### 2.2. Testing Methodology

#### 2.2.1. Research Apparatus

For the laboratory testing of the thermoelectric modules used, the methodology developed in a previous work [12] was used. It included the simultaneous measurement of both thermal and electrical parameters. The research was carried out on a test stand that will be referred to as the ‘skin model’. This stand is equipped with an electrically heated, constant-temperature measuring plate and measures the heat flow rate from the plate. The ‘skin model’ was placed in a WEISS WK11 340 microclimate chamber, which ensured constant and controlled climatic conditions during the tests. To determine the electrical power drawn by the module, its current and voltage (between the ends of the original leads) were measured.

#### 2.2.2. Research Conditions

The tests were carried out at an ambient and plate temperature of 35 °C, a relative humidity of 40% and an air movement velocity of 1 m/s. This relative humidity corresponded to those at a glassworks workplace. A constant temperature was maintained in the climate chamber throughout the test. Isothermal conditions were ensured (i.e., the temperature of the plate equaled that of the ambient air) so that heat exchange between the plate and the cold side of the thermoelectric module occurring due to the operation of the module could be measured during the actual tests, without interference from heat exchange between the plate and the environment. This made it possible to measure the cold-side heat flow rate.

#### 2.2.3. Measured and Calculated Parameters

The laboratory tests included the measurement of both thermal and electrical parameters. In terms of electrical parameters, the voltage across the TEM, U, and the current drawn by the TEM, I, were measured. On this basis, the electric power supplied to the TEM was calculated as

P_o_ = U I. (1)

In terms of thermal parameters, the power supplied to the measuring plate of the ‘skin model’, which was required to maintain a constant temperature of 35 °C at the heating plate, was measured. Due to the isothermal conditions of the test, the power supplied to the plate with the TEM power supply switched off was 0 W, so the cold-side heat flow rate of the TEM, Q_c_, equaled the power supplied to the plate. To determine the overall effect, both the electric power and the heat flow rate were averaged over the entire experiment duration for each particular value of the supply current I.

To find the maximum cold-side heat flow rate, Q_m_, the averaged Q_c_ = f(I) characteristic was approximated with a smooth second-order polynomial regression curve obtained by the least squares method using the polyfit function in Octave:

Q_c_(I) = c_Q2_ I^2^ + c_Q1_ I + c_Q0_, (2)

where c_Q0_, c_Q1_ and c_Q0_ are constant coefficients. The location of the maximum can then be calculated as

I_m_ = I(Q_m_) = −c_Q1_/(2 c_Q2_). (3)

Ultimately,

Q_m_ = Q_c_(I_m_) = c_Q2_ I_m_^2^ + c_Q1_ I_m_ + c_Q0_. (4)

Any current can then be represented as normalized with respect to I_m_:

I_norm_ = I/I_m_. (5)

To determine the coefficient of performance (COP) of the TEM, the averaged P_e_ = f(I) characteristic was also approximated with a second-order polynomial regression function with zero constant term:

P_e_(I) = c_P2_ I^2^ + c_P1_ I, (6)

with constant coefficients c_P0_, c_P1_ and c_P0_. The average COP was calculated according to

COP = Q_c_/P_e_. (7)

To ease performance comparisons of TEMs with different surface areas, the corresponding cold-side heat flux, q_c_, and electric power density, p_e_, were additionally calculated as

q_c_ = Q_c_/S, (8)

p_e_ = P_e_/S, (9)

where S is the TEM surface area. Consequently, the maximum heat flux equals

q_m_ = Q_m_/S. (10)

An unbiased estimator of the variance, σ^2^, related to a least squares fitting is [37]

s^2^ = S_rs_/ν, (11)

where S_rs_ is the residual sum of squares and ν is the number of degrees of freedom. Consequently, the standard deviation of Q_c_ calculated from (2) or that of P_e_ calculated from (6) can be evaluated as

σ = N_r_/ν^1/2^, (12)

where N_r_ = S_rs_^1/2^ is the relevant norm of residuals. Both the parameters N_r_ and ν are returned by the polyfit function [38]. The standard uncertainty of I_m_ can be evaluated by applying the rules of uncertainty propagation [39] to (3), yielding

σ(I_m_) = (I_m_/2) × {[σ(c_Q1_)/c_Q1_]^2^ + [σ(c_Q2_)/c_Q2_]^2^}^1/2^, (13)

The required standard deviations of regression function coefficients, σ(c_Q2_) and σ(c_Q1_), can be evaluated from the covariance matrix returned by the polyfit function [38]:

σ(c_Qi_) = (C_i,i_/ν) N_r_ for i = {1, 2}. (14)

As Q_c_ and P_e_ were obtained with different measuring devices, their errors are independent of each other. Hence, the standard uncertainty of the COP calculated from (7) is [39]

σ(COP) = COP {[σ(Q_c_)/Q_c_]^2^ + [σ(P_e_)/P_e_]^2^}^1/2^. (15)

As the surface area S is not subject to uncertainty, the standard uncertainties of q_c_ and p_e_ calculated from (8) and (9) equal

σ(q_c_) = σ(Q_c_)/S, (16)

σ(p_e_) = σ(P_e_)/S. (17)

Based on the latter parameter, the number of modules of a given type required to conduct a given amount of heat per unit time from the cold side, Q_c(req)_, is calculated as

M = Q_c(req)_/(q_m_ S). (18)

The actual total maximum average cold-side heat flow rate and the total surface area occupied by such a set of modules are then calculated as

Q_M_ = M Q_m_, (19)

S_M_ = M S. (20)

It should be noted that Equation (19) neglects possible differences in the heat flow between individual TEMs due to losses in interconnecting wires. The amount of these losses will depend on cooling garment construction, including the geometrical arrangement of the TEMs, their electrical configuration, and the type, length and cross-sections of wires. These parameters cannot be known before the type and number of TEMs are established, so Equation (19) is an appropriate tool to achieve this aim. The losses mentioned have to be taken into account as soon as the relevant design decisions are made.

On the other hand, the number of modules of a given type required to obtain a given total surface area, S_req_, is calculated as

N = S_req_/S. (21)

The actual total surface area and the maximum total heat flow rate that can be obtained with such a set of modules is then calculated as

S_N_ = N S, (22)

Q_N_ = q_m_ S_N_. (23)

Assuming a parallel connection of all the modules in the set, their total supply current at the maximum heat flow rate point is calculated as

I_x_ = x I_m_, (24)

where x stands for M or N, as appropriate.

#### 2.2.4. Research Procedure

For each of the selected modules, tests were conducted for TEM supply current intensities varying from 0.05 A to 0.55 A with a step of 0.05 A. To minimize the influence of variables such as the heat sink’s sorption capacity, a new heat sink was prepared and used at each particular current intensity. This enabled the tests to be carried out under repetitive initial conditions.

**Figure 2:**
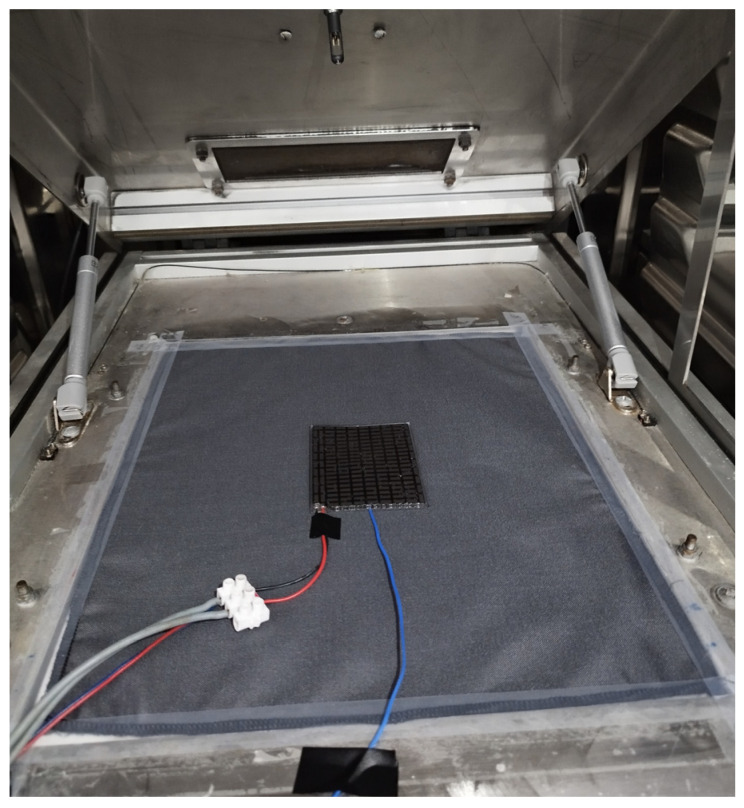
View of one of the selected thermoelectric modules placed on the multi-shield fabric on the ‘skin model’.

The tests were carried out in a multi-shield fabric arrangement, which corresponded to the conditions of the target use, where the TEMs used in the garment will not be in direct contact with the user’s skin. The TEM under test was electrically connected to a terminal block and placed in the center of the measuring plate, so that the cold side of the module was in contact with the measuring plate through the multi-shield fabric (Figure 2). Once the module was placed on the ‘skin model’, the climatic chamber was closed and the appropriate climatic conditions in the chamber were set. Before the test was started, the conditions in the chamber stabilized, as indicated by the heating power of the test plate remaining constant.

Prior to testing, each heat sink was prepared by soaking it in distilled water at a temperature of approximately 20 °C for 1 min. To ensure conditions as uniform as possible, the temperature of the water was measured before each soak of the heat sink. The thermoelectric modules were placed on the skin model for at least 30 min before the test to ensure the stabilization of the test conditions. Once the conditions in the climate chamber had stabilized, the chamber was opened, the soaked heat sink was placed on the thermoelectric module, the climate chamber was closed and the TEM power supply was activated at the set current. These operations were carried out as quickly as possible to minimize their impact on results. The duration of each test was 30 min.

## 3. Results

**Figure 3:**
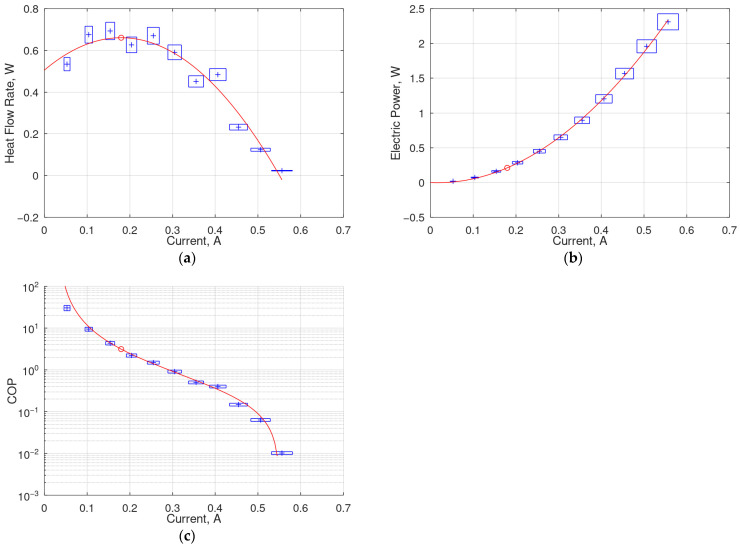
Measured (markers) and approximated (lines) characteristics of the 208 TEM as functions of the supply current, with error boxes corresponding to 3σ intervals: (**a**) average cold-side heat flow rate, (**b**) average electric power and (**c**) resulting COP; the circle marks the value corresponding to the maximum heat flow rate.

The measured and approximated characteristics of the average cold-side heat flow rate, Q_c_, and average electric power, P_e_, as functions of the supply current, I, are shown for the 208 TEM in Figure 3 together with the resulting COP calculated from Equation (7). The maximum average heat flow rate, Q_m_ = Q_c_(I_m_), as calculated from (4), together with the corresponding current, I_m_, as calculated from (3), average electric power, P_e_, as calculated from (6), average heat flux and electric power density, q_m_ = q_c_(I_m_) and p_e_, as defined by (8) and (9), and COP, as defined by (7), are indicated in Table 3 for all the tested TEMs. Their respective standard uncertainties are also included, as evaluated using Equations (12)–(17).

**Figure 4:**
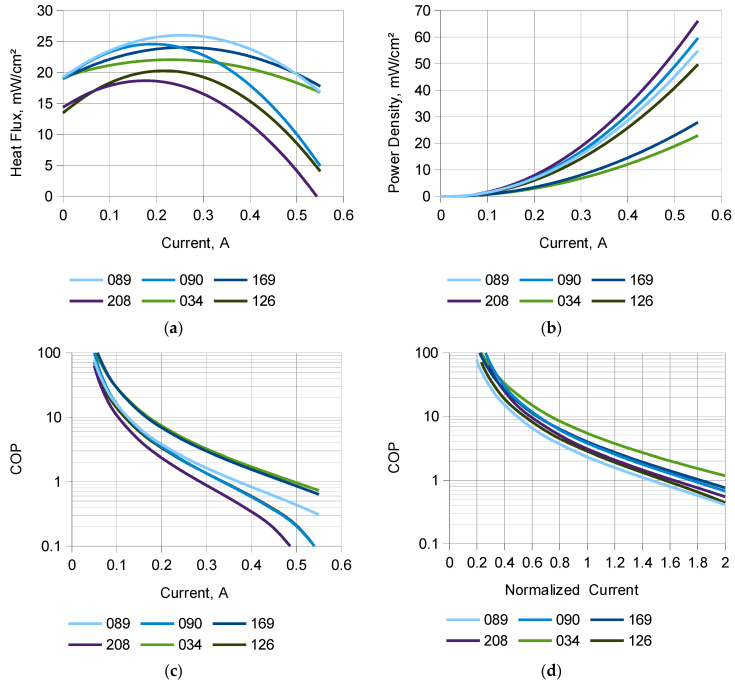
Approximated characteristics of the tested TEMs as functions of the supply current: (**a**) average cold-side heat flux, (**b**) average electric power density, (**c**) COP as a function of absolute current and (**d**) COP as a function of normalized current.

For clarity, Figure 4 presents only the approximated curves obtained for all the tested modules by least squares regression, as described in Section 2.2.3 and represented by Equations (2) and (6). To ease comparisons, instead of the heat flow rate and electric power, the heat flux q_c_ and electric power density p_e_ have been plotted, respectively. Figure 4d additionally presents the COP characteristic plotted against the normalized current I_norm_, as defined by Equation (5).

The highest maximum average cold-side heat flow rate was observed for the 169 TEM. Table 4 shows the integer number of the modules, M, of every other tested type, calculated from Equation (18), so as to obtain similar values of total heat flow rate, Q_M_, with Q_c(req)_ equal to the Q_m_ obtained for a single 169 TEM. Additionally indicated are the resulting total heat flow rates, calculated from (19), total surface area, calculated from (20), and total supply currents assuming a parallel connection of the modules, calculated from (24).

Similarly, Table 5 shows the integer number of the modules, N, of every other tested type, calculated from Equation (21), so as to obtain similar values of the total surface area, S_N_, with S_req_ equal to the surface area S of a single 169 TEM. Additionally indicated are the resulting total surface areas, calculated from (22), total heat flow rates, calculated from (23), and total supply currents assuming a parallel connection of the modules, calculated from (24). For these calculations, the 169 module was taken as a reference due its highest maximum heat flow rate, largest surface area and earlier experiments with flexible TEMs of a similar size.

## 4. Discussion

### 4.1. Supply Current, Electric Power and Heat Flux Rate

As follows from Figure 4a, the supply current I_m_ that corresponds to the maximum heat flow rate, Q_m_, is, at the same time, the maximum practical current to be applied to a TEM, because for higher currents, the heat flow rate decreases. This is because electric power, related to Joule’s heat generation, increases with current quadratically, while the heat drawn away from the hot side only increases linearly, leaving less room for heat conduction from the cold side. In a practical application, this can only be compensated for by the more efficient cooling of the TEM’s hot side. An example is the use of a heat sink, such as the one used in this work, briefly described in Section 2.1 and in more detail in [16].

By Joule’s law, the quadratic variation in power dissipation is proportional to the square of the current. However, as mentioned in Section 2.2.3 and reflected in Equation (6) and Figure 3b and Figure 4b, the power versus current characteristics contain an additional component proportional to the current. This results from the variation in the electrical resistance of a TEM with temperature, which in turn increases with increasing power dissipation and thus current. It was observed that despite this electro-thermal feedback, the TEMs were able to reach a steady condition in the range of currents applied.

It can be seen in Table 3 that the maximum current, I_m_, is similar for all the tested modules. It must be noted that due to the relative flatness of the curve in the vicinity of its maximum (especially for module 034), the uncertainty of I_m_ is quite large. The corresponding maximum average cold-side heat flux, q_m_, is between 19 mW/cm^2^ and 26 mW/cm^2^. Module 089 offers the greatest heat flux, followed by 090 and 169.

As can be observed in Figure 4a, the heat flow rate also decreases with the current decreasing below I_m_. This reflects the Peltier effect and the Fourier’s law of thermal conduction: the temperature difference created in a TEM is proportional to its current, while heat flux is proportional to temperature difference. The apparent non-linearity of the characteristic in this region also results from the quadratically varying Joule’s heat mentioned above.

It is noticeable that the approximated Q_c_ = f(I) characteristics do not cross the current axis at I = 0. This is explained by the measurement procedure, where the heat sink was initially cooler than the heating plate, leading to some heat conduction from the cold side of the TEM even with no current applied.

### 4.2. Coefficient of Performance

A coefficient of performance could be determined well based on the heat, current and voltage ratings provided by the TEM manufacturer (Table 2). However, such a value does not apply to practical systems, because it is determined for a maximum supply current and a zero temperature gradient between the hot side and the cold side. This indicates the high practical significance of the measurement results presented in this study, which were obtained in conditions that can occur in real applications.

As can be seen in Figure 4c, the COP strongly depends on the supply current. This follows from the defining Formula (7) and the behavior of its denominator and numerator, which has been explained in Section 4.1. The electric power P_e_ monotonically increases quadratically, as reflected in Figure 4b. On the other hand, the cold-side heat flow rate Q_c_ first increases, though at a decreasing rate weaker than linear, and from some point onward even decreases, which is due to the increasing Joule’s heat. Consequently, the quotient of Q_c_ to P_e_ decreases with current.

Equation (7) also explains the steep increase in the COP when the current tends to zero. Although the denominator then tends to zero as well, according to Joule’s law, the numerator is still non-zero due to the heat sink effect described in Section 4.1. It should be noted at this point that the COP values presented and discussed in this paper characterize an entire cooling system composed of a TEM coupled with a particular heat sink rather than a TEM alone.

Interestingly, third-generation modules are more favorable in terms of the COP, while second-generation ones tend to attain higher maximum heat flux values. This suggests a technology tradeoff in product development, resulting in an increased COP at the expense of a decreased maximum heat flux. Although publicly available data do not provide any details on this matter, it can be speculated that this is related to the material composition or structure of elementary thermoelectric cells. It can be observed in Figure 4b that third-generation modules not only have lower maximum heat flux values but also require lower electrical power. As electrical power is transformed into Joule’s heat, this leads to a higher COP.

Due to the COP decreasing with an increasing supply current, when it is necessary to adjust the cooling intensity (as reflected in the cold-side heat flux, q_m_), it is more efficient to supply a TEM with an appropriately set constant current than with a pulsed current with a constant amplitude and a variable duty ratio. A pulsed power supply to a TEM would involve a high current value when power is on and a zero value when it is off. Conversely, a constant power supply would have to apply an invariable current equal to the average value of the pulsed one. However, by Joule’s law, the power dissipation related to a current flowing is proportional to the rms value of that current. As the rms value involves an integration over time of the current *squared*, it would be higher for the pulsed wave due to the high-current time intervals.

Consequently, as a constant TEM current should be considered, analyzing Figure 4d provides more insight, where the x-axis represents the supply current normalized with respect to its maximum value, I_m_, for each TEM. The 034 module is the best both globally (Figure 4c) and at the maximum heat flux point (Figure 4d). However, contrary to what Figure 4c might suggest, it is followed by modules 169 and 090 rather than matched by the 126 module. Still, the relatively high uncertainty of I_m_ combined with the strong variability in the COP limits the reliability of this comparison.

The constant current requirement affects the electronic controller of the system in that filtering is necessary at its output, which increases cost and size. On the other hand, it reduces electromagnetic disturbances emitted from the system, including the wiring, thus contributing to meeting electromagnetic compatibility requirements.

### 4.3. TEM Connections, Reliability and Power Supply

The calculated numbers of modules that yield total heat flow rates or surface areas similar to the 169 module, as presented in Table 4 and Table 5, are relatively high, which can make the final product manufacturing process more difficult and costly. Moreover, the application of short (and, consequently, less flexible) wires to form interconnections between the particular TEMs, which are prone to mechanical damage, may decrease product reliability. The area necessary for the interconnections would increase the overall area occupied by the modules, while the total heat flow rate would remain unchanged, leading to a lower effective heat flux. Applying elastic textile cables or conducting threads to solve this issue is not an option as their resistances are typically higher than those of standard (metal core) wires.

Another problem resulting from connecting TEMs in parallel is the high value of the total supply current, leading to large power losses in connecting wires. Increasing their cross-section area may be impossible due to ergonomic requirements. An alternative solution would be a serial (or combined serial/parallel) connection of TEMs, but it would impose significant limitations, including the following:

- a higher required supply voltage, which would be disadvantageous for user safety;
- a lower reliability, as the failure of any one TEM might lead to the failure of the entire set (or string) either by overcurrent or open circuit.

As far as reliability is concerned, it is possible in a serial connection to detect overcurrent conditions due to a short circuit and limit the supply voltage as appropriate to protect other TEMs in the same string. One could also use additional TEM connections and power switches in the cooling system controller to bypass open-circuited TEMs, at the expense of system complexity and cost. The lifespan of the modules is not expected to be shortened even when they are operated over extended periods of time, because for each TEM tested, the maximum current I_m_ is as low as 3.0% to 4.4% of its rated current I_rat_ (cf. Table 2 and Table 3). Moreover, in the considered application, the temperatures of the TEMs will be much lower than their maximum rated temperature of 100 °C. Nevertheless, the requirement for a higher supply voltage and the related user safety issues will persist in any serial connections of TEMs.

Therefore, using a single 169 module may be the most beneficial from a practical point of view, as this minimizes the overall number of modules needed to achieve the required total heat flow rate, while providing the close-to-second-best maximum average heat flux and the second-best COP at that operating point.

The actual power requirements of a cooling system composed of the selected TEMs would depend mainly on the total number of these modules and on the electric power at the point of the maximum heat flow rate, P_e_(I_m_), as given in Table 3. Moreover, power losses in the system should be taken into account when selecting a battery to supply it. Based on earlier work on flexible TEM-based personal cooling systems [17,29], six to seven modules of dimensions similar to the 169 one are sufficient for improving the thermal comfort of humans during physical activities; the typical power loss in an electronic controller accounts for about 10% of the controller’s input power, while the power loss in interconnecting wires amounts to about 10% of its output power. This results in an average battery power consumption of 3 W for an assembly of seven 169 modules operating at their maximum cold-side heat flow rate. Hence, a 10 Ah power bank (with a nominal internal voltage of 3.7 V) would be able to supply the system for about 12 h.

## 5. Conclusions

Each specific application of TEMs imposes its own challenges. In the case of thermoelectric cooling garments, one of these challenges is the selection of an appropriate model and number of TEMs to be integrated within a garment to ensure a sufficient cooling intensity over the required surface area of the human body. It is also essential to investigate the effect of the supply current on the coefficient of performance in order to determine the optimum operating conditions for the thermoelectric module selected. To meet these needs, a methodology that enables various TEMs to be compared was developed and adopted to select the most suitable one for use in a new kind of cooling garment. Based on laboratory tests conducted for six different flexible TEM models, the heat flow rate, electric power and coefficient of performance (COP) were analyzed as functions of the supply current. This study showed that even a current variation as small as 0.05 A can result in a significant change in the coefficient of performance. It was shown that the efficiency of the modules (as represented by the COP) decreases with increasing current. The most performant module provided a heat flow rate equal to 1.39 W. Connecting thermoelectric modules with smaller surface areas but higher COPs together may prove to be less efficient in terms of their practical application, i.e., because of the reduced durability due to the interconnections. When designing cooling systems with flexible thermoelectric modules, consideration must be given to their intended use and the possibility of supplying the modules. Moreover, in order to keep the temperature difference between the sides of the module as minimal as possible, it is crucial to use an appropriate and efficient heat sink to dissipate heat from the hot side of the TEM [40,41,42]. Selecting the appropriate heat sink can significantly increase the TEM’s performance and extend its effective operating time. Therefore, new solutions for dissipating heat from the TEM’s hot side, as well as the influence of the heat dissipation method on the heat flux drawn, will be further investigated in order to optimize the TEM’s performance and the possibility of its effective use in cooling garment applications. Further work will concentrate on the use of the most performant TEM in the cooling garment, performing direct thermoelectric cooling and the evaluation of its cooling efficiency under laboratory conditions with human participants.

## Figures and Tables

**Table 1:** Parameters of various cooling systems reported in the literature.

System Type, Garment	Reference	Cooling Surface Area [cm^2^]	Heat Flux [W/cm^2^]	Δ*T*_max_ (*T*_a_) [°C]	COP (Δ*T*)	Operating Time [min]	Total Weight [kg]
Cooling liquid distribution, long-sleeve underwear	[23]	ca. 5000	ca. 0.06	1.9 (30.0)	n/a	>60	<10
Cooling liquid distribution, vest	[24]	n/a	0.12–0.18	5.7 (40.0)	n/a	120	n/a
PCM without/with fans, jacket and pants	[25]	2200	0.03/0.14	2.4/4.8 (34.0)	n/a	39/180	2.1/3.6
Vapor condenser with PCM, no garment (portable device)	[26]	n/a	n/a	n/a	>2 (n/a)	270	13
Flexible TEMs, vest	[28]	3600	0.012	>10 (33.0)	1.7 (7.0)	360 *	0.43 *
Flexible (knitted) TEMs, upper-arm band	[27]	310	n/a	0.5 (34.0)	n/a	40	n/a
Flexible TEM with RC film, on a wrist	[30]	ca. 25	n/a	9.1 (25.0)	1.6 (8.0)	39	n/a
Flexible TEM and PCM heat sink, T-shirt	[31]	n/a	n/a	>8 (32.0)	ca. 2 (3.0), ca. 4 (1.0)	>20	n/a
Flexible TEM and PCM heat sink, headband	[32]	19	n/a	4.0 (36.0)	n/a	25	n/a
Flexible TEMs and soaked heat sink, T-shirt	[16,17]	405	0.02	3.0 (35.0)	1.5 (3.0)	380	1.9
Flexible TEMs and soaked heat sink, vest	[29]	347	0.02	2.7 (25.0)	1.1–2.1 (2.7)	440	1.2
Cooling liquid distribution with rigid TEM-based radiator and fan, vest	[19,20]	38 **	5.8–7.6 **	2.7–3.2 (40.0)	3.5–4.7 (ca. 3.0)	>12.5	n/a
Rigid TEM with water cooling radiator and fan, neck band	[21]	22 **	0.45–0.68 **	ca. 10 (40.5)	ca. 1 (10.0)	30–100	0.65
Rigid TEM with air cooling, vest (tubing network for undergarment)	[22]	16 **	0.97 **	2.2 (26.1)	0.42 (2.2)	40	0.99

* estimated value; ** area of TEM that is not placed on the skin; n/a—not available; Δ*T*—drop in skin temperature due to PCS activation; Δ*T*_max_—maximum Δ*T* observed; *T*_a_—ambient temperature.

**Table 2:** Parameters of the TEGway flexible thermoelectric modules selected for tests.

Part Number	Shorthand Symbol	Technology Generation	Dimensions mm^3^	S cm^2^	I_rat_ A	U_rat_ V	P_rat_ W	Q_c(rat)_ W	q_c(rat)_ W/cm^2^
Conditions	n/a	n/a	n/a	n/a	Q_c_ = 0; ∆T_max_	Q_c_ = 0; I_max_	I_max_	I_max_; ∆T = 0	I_max_; ∆T = 0
S089A042038	089	2nd	38 × 42 × 6	16.0	6	11.6	69.6	40.2	2.52
S090A021079	090	2nd	79 × 21 × 6	16.6	6	11.6	69.6	41.6	2.51
S169A068085	169	2nd	85 × 68 × 6	57.8	6	22	132.0	76.5	1.32
S208A052068	208	2nd	68 × 52 × 6	35.4	6	27	162.0	90.6	2.56
S034A031044	034	3rd	31 × 44 × 6	13.6	6	4.4	26.4	16.8	1.23
S126A057045	126	3rd	57 × 45 × 6	25.7	6	16.4	98.4	62.3	2.43

n/a—not applicable.

**Table 3:** Electrical and thermal parameters at the maximum cold-side heat flow rate point with their standard uncertainties (in parentheses).

Module	I_m_ A	Q_c_(I_m_) W	P_e_(I_m_) W	q_c_(I_m_) mW/cm^2^	p_e_(I_m_) mW/cm^2^	COP(I_m_)
089	0.26(0.12)	0.41(0.07)	0.178(0.004)	26(5)	11.2(0.3)	2.3(0.4)
090	0.20(0.06)	0.41(0.06)	0.109(0.008)	25(4)	6.5(0.5)	3.7(0.6)
169	0.26(0.05)	1.39(0.07)	0.344(0.008)	24(2)	5.9(0.2)	4.0(0.3)
208	0.18(0.03)	0.66(0.06)	0.211(0.022)	19(2)	6.0(0.6)	3.1(0.5)
034	0.23(0.20)	0.30(0.05)	0.052(0.004)	22(4)	3.8(0.3)	5.8(1.1)
126	0.22(0.05)	0.52(0.07)	0.184(0.005)	20(3)	7.2(0.2)	2.8(0.4)

**Table 4:** Number of TEMs required to obtain a total average heat flow rate as close as possible to that of a single 169 module, together with corresponding parameters of the resulting TEM set.

Module	M	S_M_ cm^2^	Q_M_ W	I_M_ A
089	3	47.9	1.25	0.76
090	3	49.8	1.22	0.57
169	1	57.8	1.39	0.26
208	2	70.7	1.32	0.35
034	5	68.2	1.51	1.16
126	3	77.0	1.56	0.65

**Table 5:** Number of TEMs required to obtain a total surface area as close as possible to that of a single 169 module, together with corresponding parameters of the resulting TEM set.

Module	N	S_N_ cm^2^	Q_N_ W	I_N_ A
089	4	63.8	1.66	1.02
090	3	49.8	1.22	0.57
169	1	57.8	1.39	0.26
208	2	70.7	1.32	0.35
034	4	54.6	1.20	0.93
126	2	51.3	1.04	0.43

## Data Availability

The original contributions presented in this study are included in the article. Further inquiries can be directed to the corresponding author.
